# Effects of government policies on the spread of COVID-19 worldwide

**DOI:** 10.1038/s41598-021-99368-9

**Published:** 2021-10-14

**Authors:** Hye Won Chung, Catherine Apio, Taewan Goo, Gyujin Heo, Kyulhee Han, Taehyun Kim, Hakyong Kim, Yeonghyeon Ko, Doeun Lee, Jisun Lim, Seungyeoun Lee, Taesung Park

**Affiliations:** 1 Department of Chemistry, Seoul National University, Seoul, 08826 Republic of Korea; 2 Interdisciplinary Program in Bioinformatics, Seoul National University, Seoul, 08826 Republic of Korea; 3 Department of Statistics, Seoul National University, Seoul, 08826 Republic of Korea; 4 Department of Industrial Engineering, Seoul National University, Seoul, 08826 Republic of Korea; 5 Department of Archeology and Art History, Seoul National University, Seoul, 08826 Republic of Korea; 6 The Research Institute of Basic Sciences, Seoul National University, Seoul, 08826 Republic of Korea; 7 Department of Mathematics and Statistics, Sejong University, Seoul, 05006 Republic of Korea

**Keywords:** Diseases, Health care, Medical research, Risk factors

## Abstract

The outbreak of novel COVID-19 disease elicited a wide range of anti-contagion and economic policies like school closure, income support, contact tracing, and so forth, in the mitigation and suppression of the spread of the SARS-CoV-2 virus. However, a systematic evaluation of these policies has not been made. Here, 17 implemented policies from the Oxford COVID-19 Government Response Tracker dataset employed in 90 countries from December 31, 2019, to August 31, 2020, were analyzed. A Poisson regression model was applied to analyze the relationship between policies and daily confirmed cases using a generalized estimating equations approach. A lag is a fixed time displacement in time series data. With that, lagging (0, 3, 7, 10, and 14 days) was also considered during the analysis since the effects of policies implemented on a given day may affect the number of confirmed cases several days after implementation. The countries were divided into three groups depending on the number of waves of the pandemic observed in each country. Through subgroup analysis, we showed that with and without lagging, contact tracing and containment policies were significant for countries with two waves, while closing, economic, and health policies were significant for countries with three waves. Wave-specific analysis for each wave showed that significant health, economic, and containment policies varied across waves of the pandemic. Emergency investment in healthcare was consistently significant among the three groups of countries, while the Stringency index was significant among all waves of the pandemic. These findings may help in making informed decisions regarding whether, which, or when these policies should be intensified or lifted.

## Introduction

The spread of coronavirus disease^1^ in 2019 (COVID-19) became a global threat and the World Health Organization (WHO) declared it a global pandemic on March 11, 2020^2^. As of January 28, 2021, there were a total of 101,520,671 confirmed cases and 2,186,433 deaths from COVID-19 worldwide^3^.

The COVID-19 pandemic has greatly affected people’s lives, world economies and the public health threat it represents is the most serious seen in a respiratory virus since the 1918 H1N1 influenza pandemic^4^. In the absence of a vaccine or an effective treatment, the rapid spread of this disease elicited a wide range of responses from different governments across the globe to contain the spread of the pandemic. These policies were aimed at: (a) mitigation, which focuses on slowing but not necessarily stopping epidemic spread—reducing peak healthcare demand while protecting those most at risk of severe disease from infection, and (b) suppression, which aims to reverse epidemic growth, reducing case numbers to low levels and maintaining that situation indefinitely^5^. Common policies included school closures, travel restrictions, bans on public gatherings, stay-at-home orders, closure of public transportation, emergency investments in the healthcare system, new forms of social welfare provision, contact tracing, and investment in COVID-19 vaccines^6^. Transmission events occur through contacts made between susceptible and infectious individuals in either the household, workplace, school, or randomly in the community, with the latter depending on the spatial distance between contacts. Therefore, the suppression of social contact in workplaces, schools, and other public spheres is the target of such measures, which aim to reduce the transmission of the virus^7, 8^.

The effectiveness of implemented government policies in the alleviation of the COVID-19 pandemic has been demonstrated. For example, a deterministic stage-structured susceptible-exposed-infected-recovered (SEIR) model showed the positive effects of extended workplace distancing, reduction in mixing in the community, and school closure in the control of the pandemic situation in Wuhan^9^. A segmented Poisson model was used to predict the turning point, duration, and attack rate of COVID-19 in six of the G7 countries while modeling the effects of implemented policies in those countries on the spread of the pandemic^6^. The mathematical susceptible-infected-recovered (SIR) model showed the importance of “Janata curfew”, lockdowns with periodic relaxation, and workplace non-attendance used in India in curbing the spread of the virus^7^. A novel machine learning model was employed to examine the role of selected socioeconomic factors in mediating local and cross-city transmission of coronavirus in China^10^. All these studies tried to show that implemented government policies have a positive effect on reducing the spread of COVID-19.

However, a lot of the research projected the effects of these fast-changing policies by the comparison of the number of daily confirmed cases under when these policies were implemented and when not, to show the impact of these policies in the suppression and mitigation of COVID-19. The direct benefits of these policies cannot be observed but are currently only inferred from, for example, simulations or other mathematical models^11^. Therefore, a direct measure to observe if these policies had a positive impact on reducing the number of daily confirmed cases has not been done. Also, these studies have mainly focused on the effects of these actions in only a few countries like China, the USA and India^11^ but no systematic evaluation (or global effect) of these policies in mitigation of the pandemic has been made.

Our objective was to determine whether the implemented government policies had influenced the slowing down of the spread of the SARS-CoV-2 virus and observe which policies were most effective. To do this, trajectory-based analysis was carried out on 90 countries to study the relationship of the commonly used social distancing, health system, and economic policies implemented by different governments across the globe in the alleviation of the negative health, political and economic impact of the pandemic. We used the Oxford COVID-19 Government Response Tracker (OxCGRT) dataset from Blavatnik School of Government and the University of Oxford^12^. These are records of different levels of the policies in an ordinal or a numeric scale, Stringency index and indices which are arithmetic means of the different groups of policies employed by governments around the world to reduce the spread of the infection. We employed the Poison regression models using the generalized estimation equations approach^13, 14^ in the analysis of COVID-19 daily confirmed cases against policies. From this, we determined which policies had a significant influence on the number of cases deduced from the *p* values of their coefficients (significant when coefficient *p* value < 0.05). Lagging (0, 3, 7, 10, and 14 days) was also considered as intuitively the effects of policies implemented on a given day will affect the number of confirmed cases several days after its implementation.

The countries showed quite different wave patterns in their daily confirmed cases. Therefore, we grouped the countries into three groups according to the number of waves^15^ (a wave implies a rising number of sick individuals, a defined peak, and then a decline) of the pandemic observed in each country’s plot of daily confirmed cases against time in days. i.e. countries with only one wave, two waves, and three waves respectively (Supplementary Fig. 1–3). The Poisson regression model with and without lagging (a lag is a fixed time displacement in time series data), was then employed to analyze the overall relationship between cases and policies. In our first analysis, we performed the subgroup analysis for the groups of countries. This subgroup analysis assumed there is a ‘common policy effect’ across the waves observed. Through the subgroup analysis, we could identify policies that had consistently significant effects over waves. In the second analysis, we performed a ‘wave-specific’ analysis (see Methods) in which significant and effective policies may vary between the different waves of the pandemic experienced in a country. In this paper, we developed a segmentation algorithm for the identification and comparison of turning points of time series to study the spread of COVID-19 in different countries. Such turning points classify the behavior of a country’s trajectory throughout the pandemic as being in (or over) their subsequent waves^16, 17^. The goal of this wave-specific analysis was to investigate which policies had significant effects at the start of the pandemic but did not later, and vice versa. We used a 5% significance level in this analysis.

## Results

### Subgroup analysis of countries

#### Analysis of all countries

**Figure 1:**
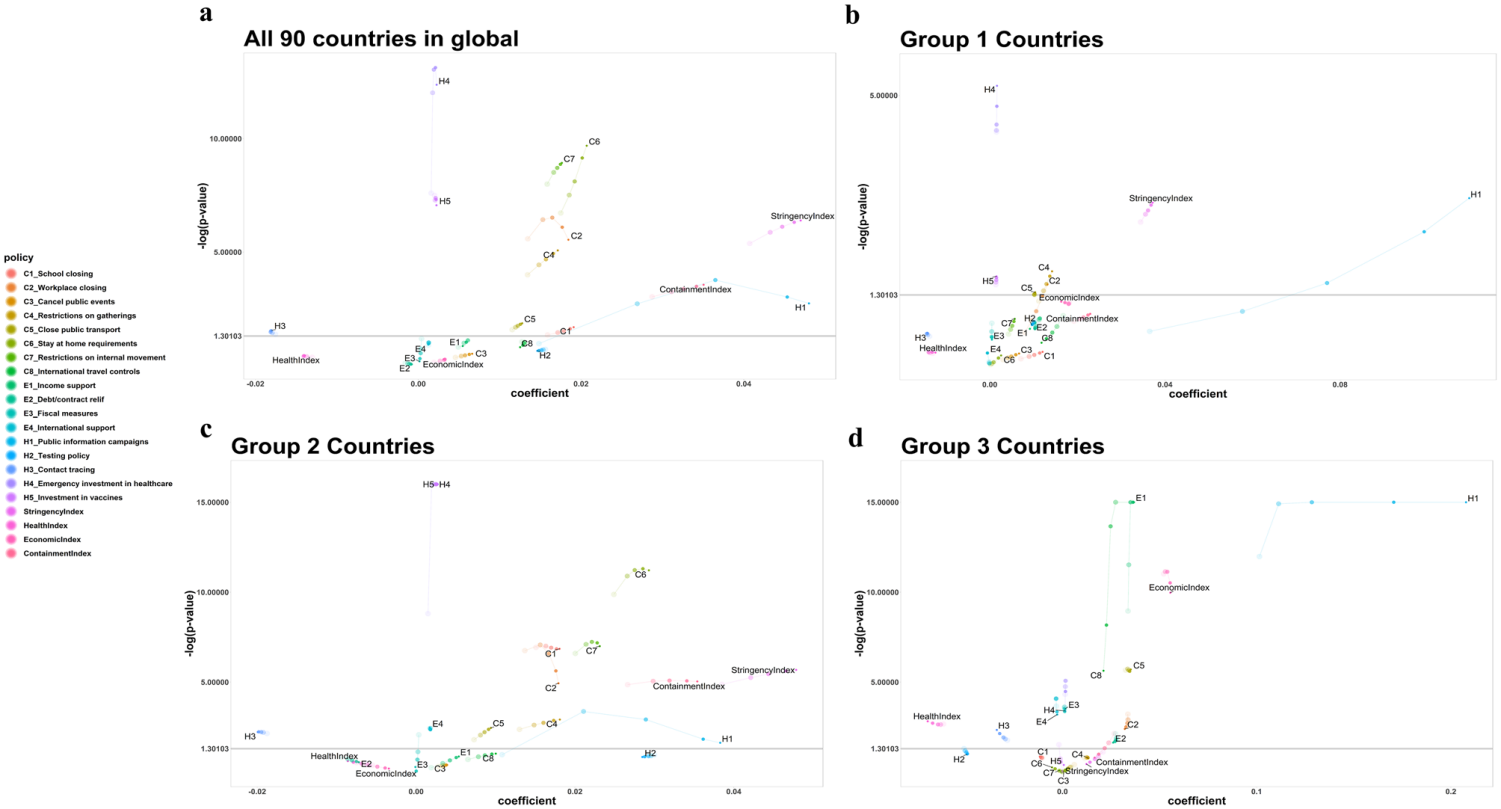
Lagging effect in the subgroup analysis. Variation of coefficients and *p* values of policies and indices according to groups is shown. The size and transparency of the circular dots increase with the time lag (0, 3, 7, 10, 14). (**a**) Analysis of all 90 countries shows C1, C2, C4, C5, C6, C7, H3, H4, H5, SI, and CoI are significant at all time lags. (**b**) Group 1 analysis shows H4, H5, and SI are significant at all-time lags. (**c**) Group 2 analysis shows C1, C2, C4, C5, C6, C7, E4, H3, H4, H5, SI, RI, CI, and CoI are significant at all-time lags. (**d**) Group 3 analysis shows C2, C5, C8, E1, E2, E3, E4, H1, H3, H4, HI, and EI are significant at all-time lags.

Figure 1 shows the variation of coefficient values and -log(*p* value) of policies. Policies with *p* value < 0.05 (− log(*p* value) > 1.30) were significant, and for a one-unit change in the predictor variable (policies and indices), the difference in the logs of expected counts (COVID-19 daily confirmed cases) is expected to change by the respective regression coefficient. A model assuming the effects of policies is the same regardless of the wave patterns was fitted without and with lagging for all 90 countries (Fig. 1a). Regardless of lagging, C1 (School closing), C2 (Workplace closing), C4 (Restriction on gatherings), C5 (Closing public transport), C6 (Stay at home requirements), C7 (Restrictions on internal movement), H3 (Contact tracing), H4 (Emergency investment in healthcare), H5 (Investment in vaccines), SI (Stringency index) and CoI (Containment index) showed significant results in their coefficients with *p* values < 0.05. However, all economic policies, C3 (Cancel public events), C8 (International travel controls), and H2 (Testing policy) were not significant irrespective of lagging. H1 (Public information campaign) was the only policy whose significance changed at the 14-days’ lag. Among the significant policies, H5 had nearly the same coefficient values while the others showed the general trend of decrease in coefficients and increase of *p* values with lagging, but only H3 had negative coefficient values (− 0.0180, − 0.0181, − 0.0181, − 0.0179, − 0.0176). However, since countries had different situations and patterns in their daily confirmed cases, countries were divided into three groups for better interpretation.

#### Analysis of Group 1 for the countries with one wave

Grouping the countries according to the number of waves in their daily confirmed cases showed that significant policies and indices varied with lagging and in the three groups. Figure 1 shows the coefficients and *p* values of policies and indices for the three groups. For the 31 countries in Group 1 (Fig. 1b), H4 (Emergency investment in healthcare), H5 (Investment in vaccines), SI (Stringency index) were significant at all-time lags and had positive coefficients. However, SI showed a trend of increasing *p* values and decreasing coefficients with lagging, whereas coefficients of H4 and H5 remained unchanged regardless of lagging. C2 (Workplace closing), C4 (Restrictions on gatherings), C5 (Closing public transport), H1 (Public information campaigns), and RI (Restriction index) all had positive coefficients and were significant only for short time-lags. H1 had a large coefficient value of 0.1 compared to those of other policies. Other policies including all economic policies were non-significant.

#### Analysis of Group 2 for the countries with two waves

In the 54 countries (Fig. 1c), C1 (School closing), C2 (Workplace closing), C4 (Restriction on gatherings), C5 (Closing public transport), C6 (Stay at home requirements), C7 (Restrictions on internal movement), E4 (International support), H3 (Contact tracing), H4 (Emergency investment in healthcare), H5 (Investment in vaccines), SI (Stringency index), RI (Restriction index), CI (Closing index), and CoI (Containment index) were significant at all-time lags. Among those, only H3 had a negative coefficient value. H3, H4, and H5 showed similar coefficients and *p* values with lagging whereas others showed a similar trend of increasing *p* values and decreasing coefficient values with lagging. E3 (Fiscal measures) became significant while H1 (Public information campaigns) became non-significant at 14-days’ lag with both having positive coefficients values.

#### Analysis of Group 3 for the countries with three waves

In the 5 countries (Fig. 1d), C2 (Workplace closing), C5 (Closing public transport), C8 (International travel controls), E1 (Income support), E2 (Debt/ contract relief), E3 (Fiscal measures), E4 (International support), H1 (Public information campaigns), H3 (Contact tracing), H4 (Emergency investment in healthcare), HI (Health index), and EI (Economic index) were significant regardless of lagging. Of these, E4, H3, and HI had negative coefficients. The patterns of coefficients and *p* values varied across policies. An interesting trajectory was observed with H1: at the 14-days’ lag, its *p* value suddenly increased, and the coefficient decreased greatly compared to the other policies. H2 (Testing policy) and H5 (Investment in vaccines) became significant at 14-days’ lag but their coefficients were near 0 while CoI (Containment index) became significant at larger lagging days (see Fig. 4a for variation of only indices across groups).

### Wave-specific analysis

Wave analysis (see Methods) simultaneously fits all 90 countries and considers wave information for all the countries without the need for grouping as performed under subgroup analysis. Coefficients and *p* values for the first wave were obtained from all countries since all countries experienced the first wave of the pandemic. Coefficients and *p* values of second and third waves were calculated from only countries that experienced those waves. This analysis was performed to determine whether significant policies differed according to waves of the pandemic experienced among the countries. Comparison of coefficients and *p* values for the policies and indices for the first wave, second wave, and third wave at all-time lags showed that different indices and policies were significant depending on the wave as described below.

#### Analysis of the first wave

**Figure 2:**
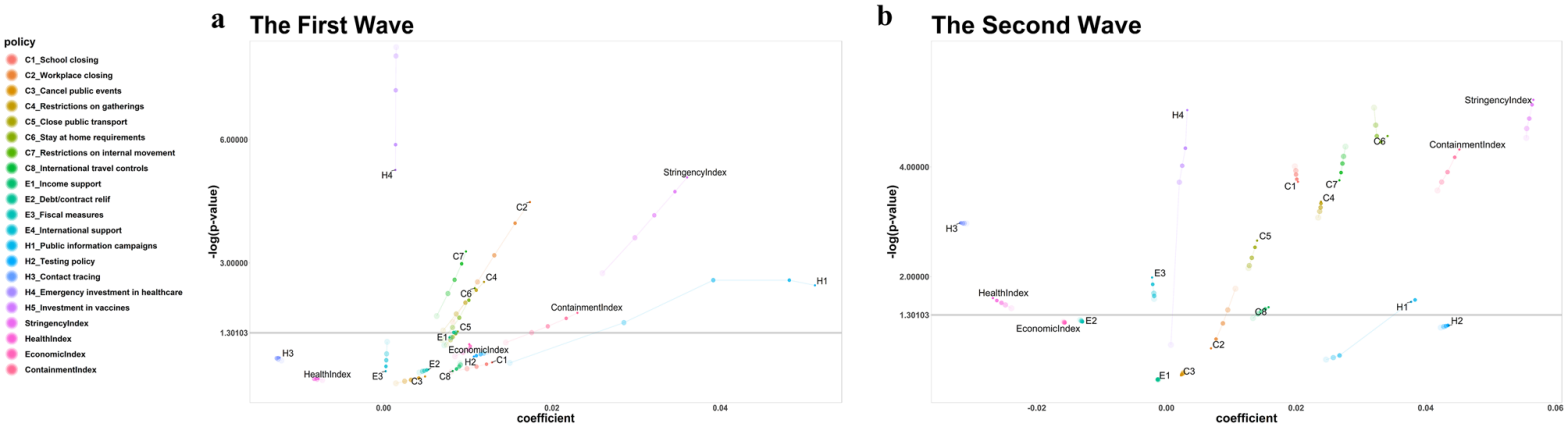
Lagging effect in the wave-specific analysis. Variation of coefficients and *p* values of policies and indices in the first wave analysis is shown. The size and transparency of the points increase with a time lag (0, 3, 7, 10, 14). (**a**) first wave analysis shows C2, C4, C7, H4, SI, and RI were significant at all-time lags while C8 and E3 were with lagging only. (**b**) second wave analysis shows C1, C5, C6, E3, H1, H3, HI, CI and CoI are significant at all-time lags while C8 at 14 days’ lag.

For the first wave analysis (Fig. 2a), C2 (Workplace closing), C4 (Restrictions on gatherings), C7 (Restrictions on internal movement), H4 (Emergency investment in healthcare), SI (Stringency index), RI (Restriction index) were significant regardless of lagging. C8 (International travel controls), E3 (Fiscal measures) became significant with lagging. All significant coefficients were positive. H1 (Public information campaigns), HI (Health index), CoI (Containment index) became non-significant after 14-days’ lag. The trend of increasing *p* values and decreasing coefficients was also observed with significant indices, closure, and containment policies (C policies). H4 (Emergency investment in healthcare) showed a decrease in *p* values while the coefficients stayed similar.

#### Analysis of the second wave

For the second wave (Fig. 2b), C1 (School closing), C5 (Closing public transport), C6 (Stay at home requirements), E3 (Fiscal measures), H1 (Public information campaigns), H3 (Contact tracing), HI (Health index), CI (Closing index), CoI (Containment index) were significant at all-time lags while H3 and HI had negative coefficients. H4 (Emergency investment in healthcare), C8 (International travel controls) became non-significant after 14-days’ lag and H1 (Public information campaigns) was significant in smaller lagging days but became insignificant in larger lagging days. Moreover, H1 showed a large difference in coefficients with lagging, and it showed a larger positive coefficient in smaller days lagging.

#### Analysis of the third wave

**Figure 3:**
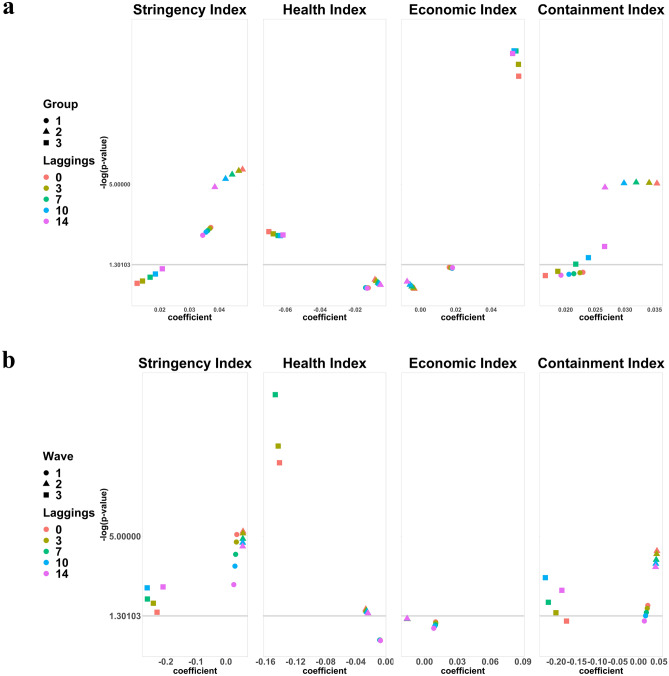
Subgroup and wave-specific analysis results for the 4 indices. This figure shows the variation of coefficients values and *p* values of only indices. Each group (or wave) is represented by different shapes. Circle, triangle, and square represent Group 1, 2, and 3 countries (or first, second, and third wave), respectively. The colors represent the time lags. (**a**) Show the variation of coefficient values and *p* values of indices from the subgroup analysis. (**b**) Shows the variation of coefficient values and *p* values of indices from the wave-specific analysis.

For the third wave, C1 (School closing), C3 (Cancel public events), C5 (Closing public transport), C6 (Stay at home requirements), C8 (International travel controls), E1 (Income support), E3 (Fiscal measures), H3 (Contact tracing), HI (Health index), CI (Closing index), EI (Economic index) were significant with all coefficients near 0 while HI had a negative coefficient (see Fig. 3b for variation of coefficient values and -log(*p* values) for the four indices across wave-specific analysis).

**Figure 4:**
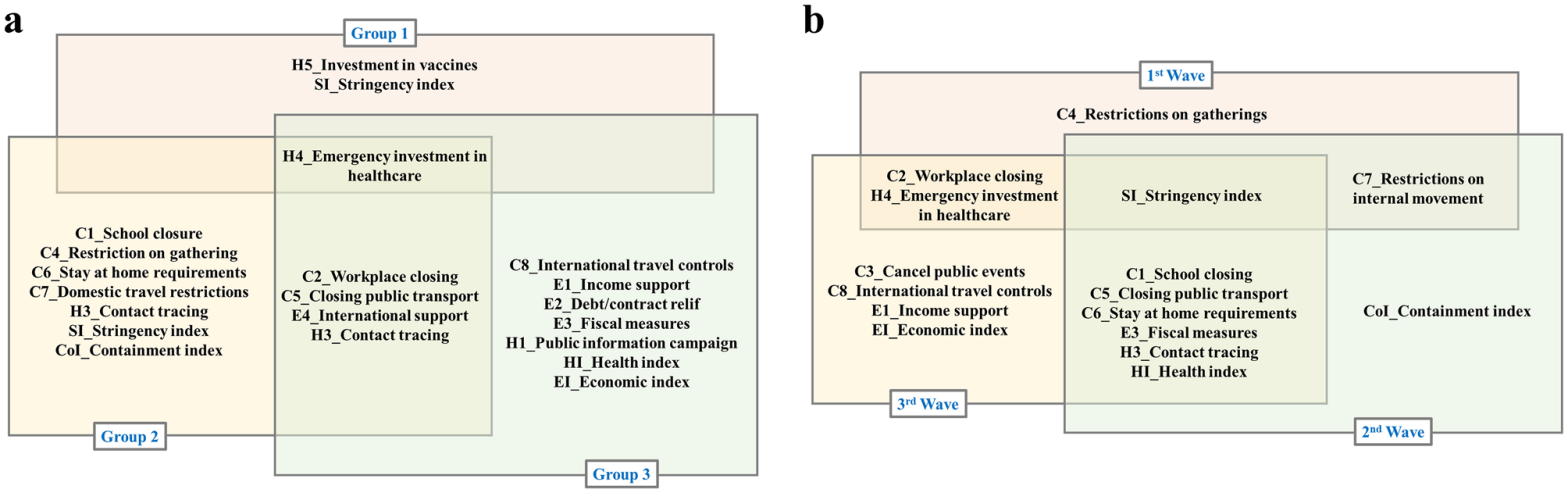
Venn diagram of significant policies at all time lags. (**a**) Significant policies and indices from subgroup analysis and (**b**) show significant policies and indices from the wave-specific analysis at all-time lags.

For comparison purposes, significant policies regardless of lagging for subgroup analysis and wave-specific analysis are shown in the Venn diagrams below (Fig. 4). In subgroup analysis (Fig. 3a), Group 2 had more significant policies followed by Group 3, then Group 1, while in wave-specific analysis, third wave analysis had more significant policies followed by second wave and then first wave analysis (Fig. 3b). Group 1 had countries that experienced or were experiencing the first wave of the pandemic as of 31st August 2020, while the first wave analysis showed that all closing and restriction policies (the C policies) were significant, which can be interpreted that at the start of the pandemic, many countries may have enforced these policies as the first response to the new disease. For Group 2 countries and second wave analysis, we observed the impact of contact tracing and closing policies on the number of confirmed cases that could not be observed during the first wave and with Group 1. Group 3 and third wave analysis brings economic policies in addition to the other policies being significant. In addition, H4 (Emergency investment in healthcare) was significant in all groups while the Stringency index which is the overall strictness of all policies was significant in all waves.

## Discussion

A look at history tells that pandemics have consistently been part of human history, and governments have continually implemented a variety of policies in their response, such as quarantines during the Ebola oubreak^18, 19^. While most of these actions have proven effective in past infections, they have always had high social and economic costs on the people. Our results provided evidence that the implemented government policies had an impact on reducing the number of COVID-19 daily confirmed cases. Also, for the Poisson regression models, result interpretation was centered around the coefficients of the predictor variable.

H3 (Contact tracing) and the Health index (HI) had consistent negative coefficients across all analyses. Therefore, this can be interpreted that intense testing and accurate tracing of infected persons and contact tracing were paramount in the reduction of the number of COVID-19 daily confirmed cases. Intuitively, if the strictness of policies and daily confirmed cases had a negative correlation, it provides theoretical support for the reason why governments implemented stricter policies despite the inconveniences^19–22^. However, most of the significant policies had positive coefficients. This may be because the daily confirmed cases and policies may be correlated in more complex ways or it shows the impact of other factors not considered here on the spread of COVID-19, which needs to be addressed. For example, daily confirmed cases dramatically increase due to unexpected aspects regardless of the policies. When this happens, governments usually apply stricter policies to decrease the number of confirmed cases. However, the daily confirmed cases usually do not decrease immediately after the implementation of these policies. Instead, the increased rate of daily confirmed cases may decrease. This was shown by the general trend of decreasing coefficients with increased lagging observed with many of the policies. This shows that policies require some time before their influence on the spread of COVID-19 can be observed. Therefore, in the perspective of treating policy as the cause and confirmed cases as the effect, our result can be confusing, however, in the perspective of interpretation of the phenomenon itself, the results are meaningful.

A more general analysis is possible in Groups 1 and 2 than in Group 3 since there were only 5 countries in Group 3 and the results can explain trends of those countries but not for others. A general trend of decrease in coefficients with larger lagging was observed for many policies except with H1 (Public information campaign). H1 consistently showed better significance (smaller *p* values) in smaller lagging days. This indicates that H1 is the type of a policy whose effects are immediately observed after implementation and after 14 days, the effect turns out to be statistically insignificant. Considering the large positive coefficient compared to other policies, we can think that public campaigns began immediately as a response to the new pandemic or increase in daily confirmed cases. Thus, it shows a large positive correlation, however, after almost 2 weeks it is hard to find a direct effect of this policy alone. In addition, H4 (Emergency investment in healthcare)—buying of ventilators and other quarantine equipment, drugs, mask donation, setting up of local testing stations—was significant in all groups. This suggests that emergency investment in healthcare helped in the prevention of overwhelming of the healthcare system hence mitigation of the pandemic.

Lastly, wave-specific analysis was performed to observe if the effects of policies differed depending on which wave the policies are enforced in. In the first wave of the pandemic, most of the policies directly related to closure and containment (C policies) were significant with a trend of decreasing coefficient values with lagging. During the first wave of the pandemic, since much was not yet known about the epidemiological characteristics of COVID-19. Thus, most countries may have focused on closure and containment policies, therefore their effects are well observed. For the second wave, various closing and containment policies became significant, in addition to Health index and one economic policy E3 (Fiscal measures). Even though it is hard to interpret how the economic policies affect the daily confirmed cases, we observed that many countries became aware of the importance of economic policies during the second wave. Especially, E3 (which is announced economic stimulus spending and included any spending or tax cuts not included in E4, H4, or H5) had negative coefficients. This can be interpreted that monetary help from the government for businesses or living expenses may have motivated people to comply with stay-at-home orders, lockdowns, or working from home which led to fewer confirmed cases. H3 and this time Health index (which shows overall strictness of health-related policies) had negative coefficients. This shows the effectiveness of contact tracing and other health policies in decreasing the daily number of confirmed cases. Moreover, the Stringency index (which is a measure of the overall strictness of the policies) was significant in all waves. Even though significant policies differed across waves, the Stringency index always showed their consistent effect across all waves.

As of when this paper was written, more countries were experiencing the third wave of the pandemic, and some were starting their fourth wave. Also, newer policies have been included in the OxCGRT Policy Dataset like H7 (Vaccination Policy), H6 (Facial Coverings) and people’s attitudes towards these policies in some of the countries have changed drastically. In the future, we hope to continue this analysis with consideration to newer waves of the pandemic and policies. Moreover, some data with negative cases have been treated as zeros (see Methods) for the analysis. Alternatively, we can treat the negative counts (or values cases) by the following three methods; (1) replace the negative values with the mean the day before and the day after, (2) distribute the negative value equally to the number of cases during 1–2 weeks before, or (3) apply the *Savitzky—Golay* filter to transform the negative values to the positive values^16^. However, they all have shortcomings and require more investigation for future studies.

In conclusion, with the current results, we observe a few direct negative relationships between the daily confirmed cases and some policies (or indices), making it difficult to present a straightforward overall interpretation of the results of this analysis. However, the results still have great meaning; the relationships between implemented policies and confirmed cases with lagging and groups or waves of this new pandemic were explored. In addition, the analysis was done till August 31st, which is the end of the summer and by this time enough second-wave countries existed. In further research, the analysis date can be extended and compared to how the confirmed cases’ pattern has changed and fit the respective GEE approach for analysis. This may give more insight into the significance of policies at different waves of the pandemic. Furthermore, the pandemic is still ongoing, and many experts are expecting the pandemic will continue even after the vaccine^23, 24^. Thus, this research including defining breakpoints and fitting GEE models needs update until the declaration of the end of the pandemic. Nevertheless, it still can serve as the first trial to investigate the effect of policies in the global perspective rather than only encountering a small group of or selected countries.

## Methods

### COVID-19 data

The COVID-19 data of daily confirmed cases and deaths can be downloaded from the European Centre for Disease Prevention and Control (ECDC) website^3, 25, 26^. Negative confirmed cases were corrected to 0 regarding it as an abnormal count. The data of 90 countries from December 31, 2019, to August 31, 2020, with a total of 26,240,426 confirmed cases and 866,354 deaths worldwide, was used in the analysis.

Data smoothing is used to remove noise from a data set, allowing important patterns to stand out. Thereafter, daily confirmed case data was smoothed by simple moving average; 1) to reduce the effect of outliers and 2) remove the weekly periodicity observed in the data. Several outliers showed greater or smaller abnormalities, which made it difficult to fit the statistical model. In addition, weekly periodicity was observed in the daily confirmed case data for many countries. Although we tried to present numerically through autocorrelation function, the trend also had randomness, so there was a limit to the analysis. Therefore, considering 7 days, the window size was set to 7 and simple moving average (SMA) was used.

### OxCGRT policy data

The ordinal and numeric dataset of policies is from the Blavatnik School of Government and the University of Oxford called the Oxford COVID-19 Government Response Tracker (OxCGRT) dataset^12^. OxCGRT systematically collects information on several different common policy responses that governments have taken to respond to the pandemic on 17 indicators (Text type policy excluded from this analysis), from more than 160 countries. Eight of the policy indicators (C1-C8) record information on containment and closure policies, such as school closures and restrictions in movement. Four of the indicators (E1-E4) record economic policies, such as income support to citizens or provision of foreign aid. Five of the indicators (H1-H5) record health systems policies such as the COVID-19 testing regime or emergency investments into healthcare. The data from the 17 policies are aggregated into a set of four common indices, reporting a number between 1 and 100 to reflect the level of government action on the topics in question; (1) a Containment index (which combines ‘lockdown’ restrictions and closures), (2) Health index (with measures such as testing policy and contact tracing, short term investment in healthcare, as well investments in vaccines), (3) an Economic support index (which records measures such as income support and debt relief), (4) as well as the original Stringency index provided in OxCGRT (which records the strictness of ‘lockdown style’ policies that primarily restrict people’s behavior)^27, 28^. The Containment index includes the eight C policies (C1–C8) associated with the closure and containment, while the Stringency index includes the eight policies plus H1(public information campaign). Although it is quite redundant, the Stringency index was included in the analysis since it was a representative index already defined by the OxCGRT.

For numerical types of data, log transformation with base 10 was done. To adjust the differences between policies, ordinal data was divided by a possible maximum value of each data. Then it was multiplied by 100 thus having data range from 0 to 100. Policies that apply only to a subunit of the given jurisdiction (for example, a single state of a country being coded) are flagged as “targeted”, while policies that apply to the whole jurisdiction are flagged as “general”^29^. For example, the ordinal scale of C1 (School closing) presents information about the strictness of the policy: 0 for no measures, 1 for recommend closing, and 2 for require closing. Additional information, the flag, indicates whether the policy is implemented either “targeted” or “general”. If school closure is recommended for some regions of the country, the ordinal scale and the flag will be 1 and 0 respectively. If school closure is required for all schools in the country, the ordinal scale and the flag will be 2 and 1 respectively. Similar descriptions can be found for other policies except for E1. In the case of E1, the flag is determined by whether income support is only done for formal sector workers or both formal and informal sector workers.

Therefore, if the policy had a flag, it was treated with an additional level of 1 for the policy while the maximum value was also increased by 1. To rescale all policies into values ranging from 0 to 100, the possible maximum value of each policy should be calculated beforehand. Thus, we set the maximum value as (maximum ordinal scale) + 1 to consider the flag for policies with flag information. If a policy has a flag, it was treated with an additional level of 1 for the strictness of the policy. For example, E1 has the ordinal scale of 0, 1, 2 but since it has a flag, its maximum value was calculated as 3.

1 $$Index=\frac{policy\,\,level}{\mathit{max}\,\,level} (\mathrm{without\,\,flag})$$

2 $$Index=\frac{policy\,\,level+flag}{\mathit{max}\,\,level+1} (\mathrm{with\,\,flag})$$

where the max level is the maximum ordinal value or numerical value for a given policy, policy level is the ordinal value or numerical value being implemented plus flag (0 or 1).Since ordinal scales or numeric values differ among policies, the index value is calculated before rescaling. ‘Max level’ in the equation corresponds to the maximum ordinal value or numerical value that a policy can have. 1 is added for policies with flags and 0 vice versa.

In addition to the policies, indices which are the arithmetic mean of the relevant policies are calculated; these are defined as the composite measure of the policies. Four indices are Health index (HI; H1, H2, H3, H4, H5), Economic index (EI; E1, E2, E3, E4), Containment Index (CoI; C1-C8), and Stringency index (SI; C1-C8, H1) provided by OxCGRT which shows an overall measure of the strictness of government response. HI, EI and CoI were defined as the arithmetic mean of policies with the same alphabet code provided by OxCGRT.

### Poisson regression model for the subgroup analysis of countries

To determine whether the adopted government policies have been effective in mitigating and suppressing COVID-19 in different countries across the globe, we implemented a Poisson regression model^27, 28^ using the generalized estimation equation (GEE) approach^13, 14^. The Poisson model successfully predicted the COVID-19 daily confirmed cases which motivated us to employ it in our analysis^6^. The GEE approach has been widely used to analyze longitudinal data and repeated measurements. The GEE approach allows parameters’ estimation from a generalized linear model (GLM) with possible unknown correlations between the outcomes. Parameter estimates from the GEE approach are consistent even though the covariance structure is not specified, under mild regularity conditions. The GEE approach focuses on estimating the average response over the population ("population-averaged" effects) rather than the regression parameters that would enable the prediction of the effect of changing one or more covariates on a given individual^30^. Individual COVID-19 daily confirmed cases for each country showed diverse wave^15^ patterns in their daily confirmed cases. Therefore, only 90 countries were divided into three groups (Supplementary Table 2) by the number of waves of the pandemic experienced in those countries, and the Poisson model is fitted for each group, respectively. Group 1 (31 countries): countries with only one wave (Supplementary Fig. 1), Group 2 (54 countries): countries with first and second waves of the pandemic (Supplementary Fig. 2), and Group 3 (5 countries): countries with 3 waves (Supplementary Fig. 3) and analyzed using Eq. (3). Lagging ($\Delta \mathrm{t})$ was also introduced (0, 3, 7, 10, and 14 days) since intuitively, the policies will bring significant changes a few days after its implementation. In other words, daily confirmed cases of the day (t) are determined by policies of the day (t-$\Delta \mathrm{t}$). For the $ith$ country, the Poisson regression model is defined as follows:

3 $$\mathrm{log}\left({\upmu }_{\mathrm{i}}\left(t\right)\right)={\beta }_{0}+{\beta }_{1}t+{\beta }_{2}\mathrm{log}t+{\beta }_{3}{Z}_{i}\left(t-\Delta t\right),$$

where ${\mu }_{i}=E\left[{Y}_{i}\right]$, ${Y}_{i}\left(t\right)$ are the daily COVID-19 confirmed cases, *t* is the number of days since the first case, ${\beta }_{o}, {\beta }_{1}, {\beta }_{2} and {\beta }_{3}$ are regression coefficients,${Z}_{i}(\mathrm{t})$ s are the different government implemented policies from the OxCGRT (Supplementary Table 1) and (t = ∆t, ∆t + 1, …) for each policy or index variable.

### Segmentation process

Segmentation is a method of finding local maxima (peaks) and local minima (breakpoints)^16^, where a peak is the timestamp at which daily new confirmed case is highest in a segment and breakpoint is the timestamp that splits the consecutive two segments in a smoothed time series dataset using the Nadaraya-Watson kernel regression Estimator (NWE) with Gaussian kernel^31–33^.

Let ${Y}_{t}$ be the $t$-th daily new confirmed cases from data, $\widehat{f}(t)$ be the estimated $t$-th daily new confirmed cases using the above NWE since December 31, 2019. Peak detection utilizes the first and second derivative test to find local maxima on a convex function. $\widehat{f}(t)$ is concave down when $t$ is around the peak due to the nature of epidemic dynamics. Considering daily new confirmed cases being discrete time series data, we find the location where the first difference is zero and the second difference is negative (Since $f(t)$ is not differentiable, we used difference operator instead of derivative):

4 $$\Delta \widehat{f}\left(t\right)=0,{\Delta }^{2}\widehat{f}(t)<0$$

where $$\Delta \widehat{f}(t)=\widehat{f}(t+1)-\widehat{f}(t)$$ and $${\Delta }^{2}\widehat{f}(t)= \Delta \widehat{f}(t+1)-\Delta \widehat{f}(t)$$.

And for discontinuity and small variances of $\widehat{f}(t)$, we used the following conditions:

5 $$\Delta \widehat{f}\left(t\right)\Delta \widehat{f}\left(t+1\right)\le 0,{\Delta }^{2}\widehat{f}(t)<-c\cdot {argmax}_{t\in T}|{\Delta }^{2}\widehat{f}(t)|$$

where $c\in (0, 1)$ is sensitivity level and $T$ is the set of time indices from December 31, 2019, to August 31, 2020. Three additional conditions ((a) Exclusion of small peaks, (b) Resolution criteria, and (c) Exclusion of peaks which are vibrations on increasing trend) are used in peak detection to enhance robustness. After all the peaks are found, breakpoints can be defined. Supplementary Fig. 4 visualizes the segmentation process applied to USA’s COVID-19 daily new confirmed cases, where detailed descriptions on $\widehat{f}(t)$, $\Delta \widehat{f}\left(t\right),\mathrm{ and }{\Delta }^{2}\widehat{f}\left(t\right)$ are given. Detailed description on the segmentation algorithm can be found here^34^.

### Poisson regression model for the wave-specific analysis

As related in other works^16, 17^, breakpoints or segments were defined if one of the following conditions was satisfied. 1) the day with the minimum confirmed case between two peaks and 2) the with the minimum confirmed case between the last peak and last day when the number of COVID-19 confirmed cases increases continuously. The initial point of analysis was set to the first day with more than or equal to 50 daily confirmed cases since days with a small daily number of confirmed cases make the process of smoothing and model fitting difficult. The first wave is considered from the initial point of analysis to the first breakpoint, the day with the minimum confirmed case between first and the second wave (for countries with one wave only, the first wave is defined until August 31, 2020), while the second wave is considered from first breakpoint to the second breakpoint (the day with the minimum confirmed case between the second wave and the start of the third wave) and the third wave is from second breakpoint to the with the minimum confirmed case between last peak and last day. This wave information was analyzed via dummy variables using the principles presented in segmented or piecewise regression models^35, 36^ using the GEE approach, as shown below.

6 $$\mathrm{log}\left({\mu }_{i}\left(t\right)\right)={\beta }_{0}+{\beta }_{11}\left({I}_{1}*t\right)+{\beta }_{12}\left({I}_{2}*t\right)+{\beta }_{13}\left({I}_{3}*t\right)+{\beta }_{21}\left({I}_{1}*\mathrm{log}t\right)+{\beta }_{22}\left({I}_{2}*\mathrm{log}t\right)+{\beta }_{23}\left({I}_{3}*\mathrm{log}t\right)+{\beta }_{31}\left({I}_{1}*{Z}_{i}\left(t- \Delta \mathrm{t}\right)\right)+{\beta }_{32}\left({I}_{2}*{Z}_{i}\left(t-\Delta \mathrm{t}\right)\right)+{\beta }_{33}\left({I}_{3}*{Z}_{i}\left(t-\Delta \mathrm{t}\right)\right)$$

The Poisson model (4) contains interaction terms in days, log days, and the policies and thus has an underlying assumption that each wave has different coefficients for days, log days, and policies. Indicators I_1_, I_2_, I_3_ correspond to the first, second, and third waves respectively. This model allows waves to have different patterns and effects of policies. Variables *t* and *log t* were considered in the above models due to the successful implementation of the segmented Poisson model in estimating the daily confirmed cases in COVID-19 including the turning points^6^. The policies are considered significant if the *p* values are smaller than 0.05. i.e. having an impact on the daily confirmed cases of the countries used in this analysis. From the 182 countries in the OxCGRT data, 90 countries were left after excluding countries with missing values in the OxCGRT dataset, non-clear breakpoint information, and those whose periodicity patterns could not be smoothed by the above mentioned SMA method.

Since the analysis was aiming for general trends and the impact of these policies on daily confirmed cases, the GEE approach was fitted using only the ‘independence’ correlation structure. It is well known that other correlation structures such as ‘exchangeable’ and ‘AR-1’ provide approximately similar coefficients due to the robustness of the GEE approach^37^. However, coefficients were not fitted for these structures due to the large dimension of the dataset brought about by the number of many countries considered in this analysis. All analyses were implemented in R (version 3.6.3).

## Supplementary Information

Supplementary Information 1.

Supplementary Information 2.

Supplementary Information 3.

Supplementary Information 4.

Supplementary Information 5.

Supplementary Information 6.

Supplementary Information 7.

## Data Availability

The datasets analyzed during this study are daily updated public data available at https://www.ecdc.europa.eu/en/publications-data/download-todays-data-geographic-distribution-covid-19-cases-worldwide and https://www.bsg.ox.ac.uk/research/research-projects/coronavirus-government-response-tracker.
