# Supplementary figures and images for: Effects of government policies on the spread of COVID-19 worldwide
Source: Sci Rep. 2021 Oct 14;11:20495. doi: 10.1038/s41598-021-99368-9 (PMC8516948; doi:10.1038/s41598-021-99368-9)

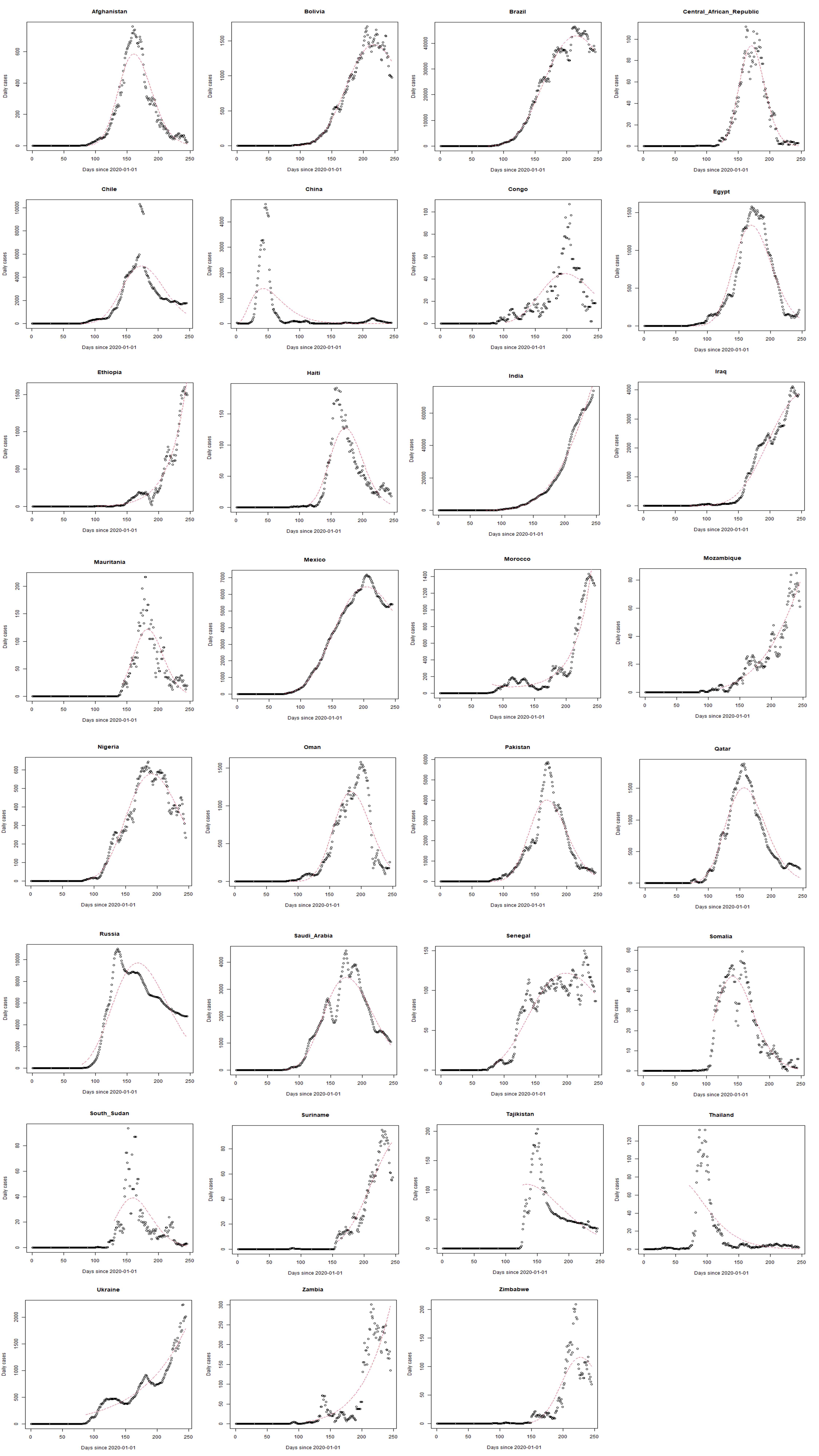

Supplement: Supplementary file 1 — Supplementary Information 1. [file 41598_2021_99368_MOESM1_ESM.png]

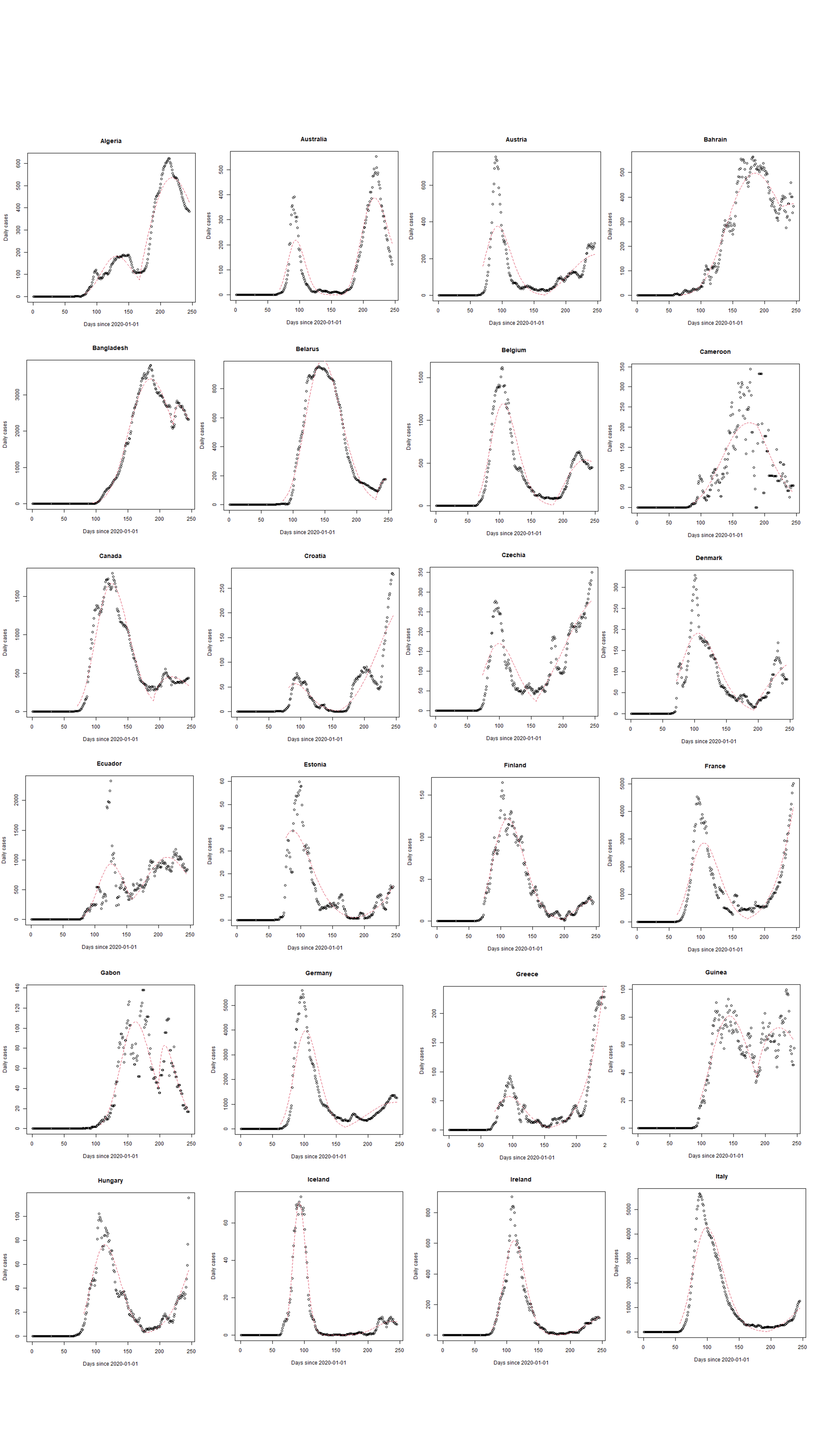

Supplement: Supplementary file 2 — Supplementary Information 2. [file 41598_2021_99368_MOESM2_ESM.png]

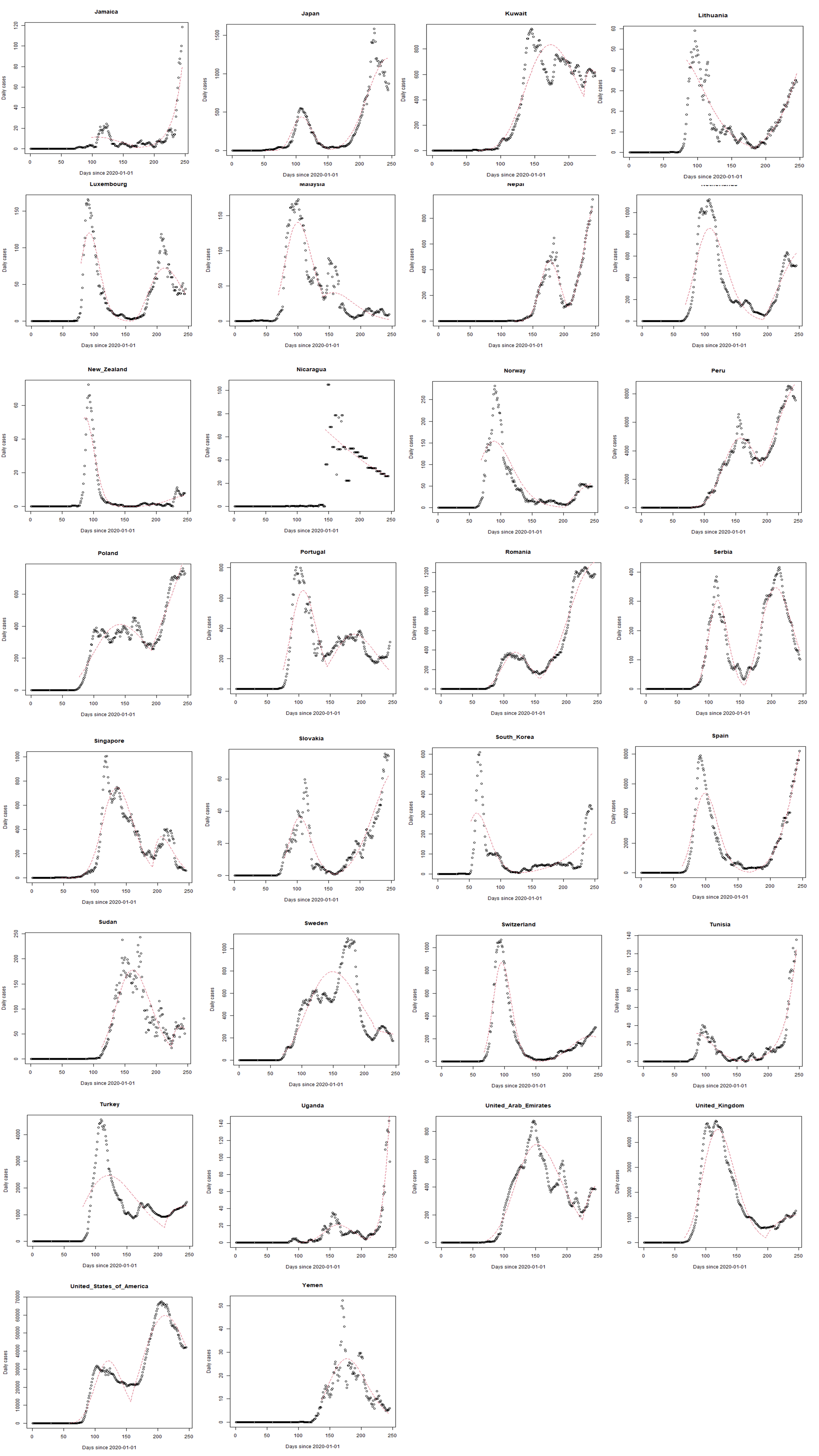

Supplement: Supplementary file 3 — Supplementary Information 3. [file 41598_2021_99368_MOESM3_ESM.png]

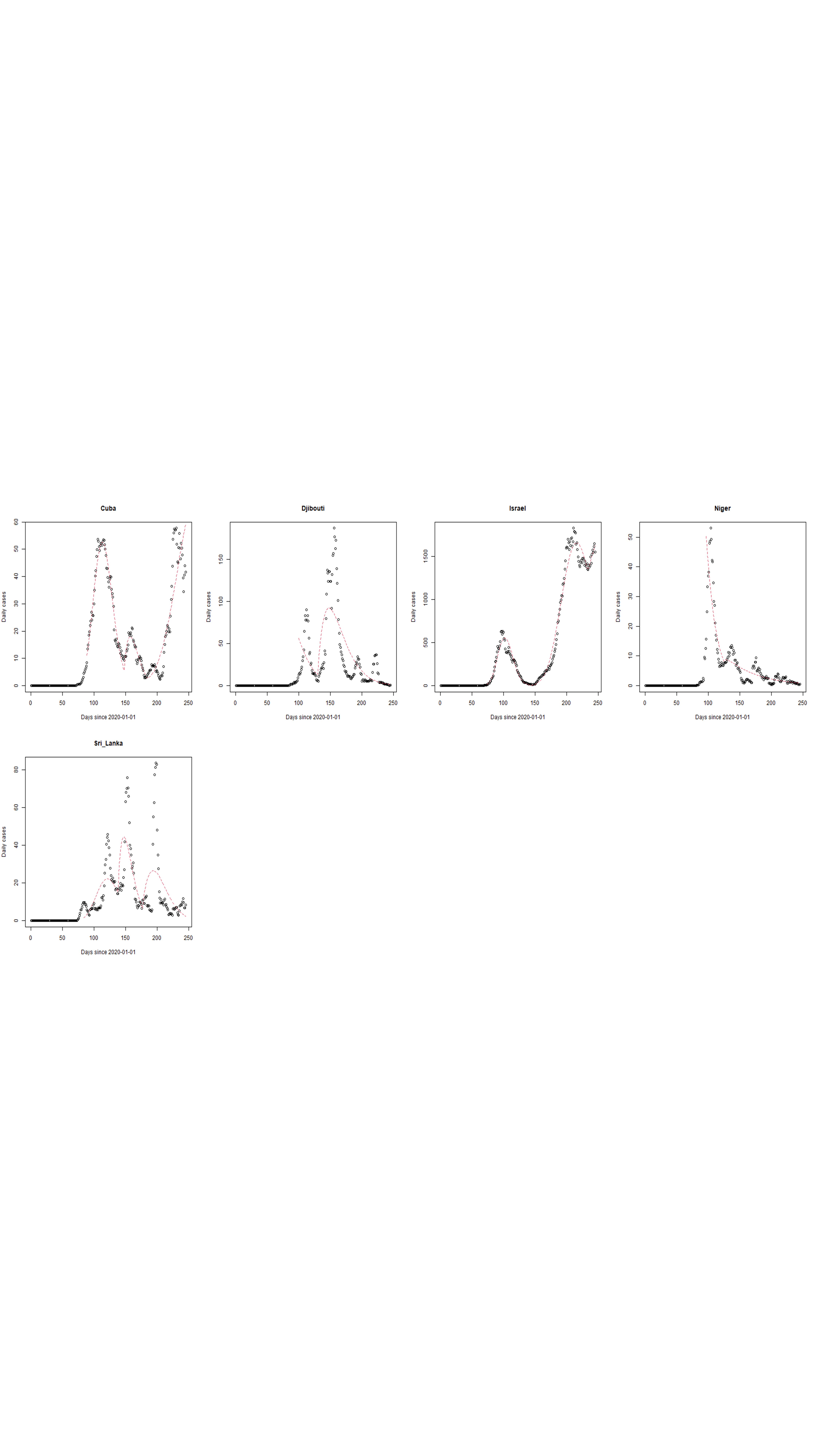

Supplement: Supplementary file 4 — Supplementary Information 4. [file 41598_2021_99368_MOESM4_ESM.png]

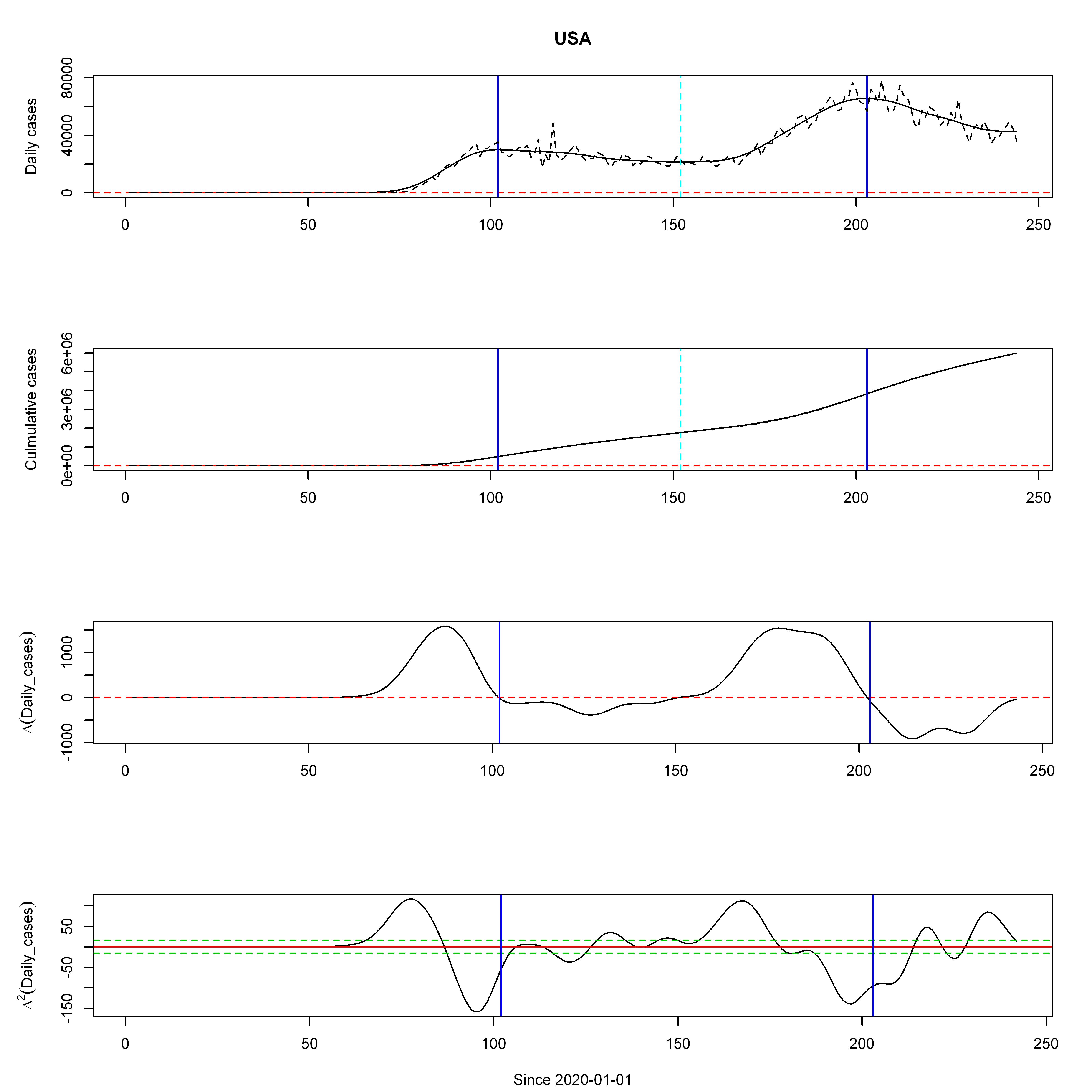

Supplement: Supplementary file 5 — Supplementary Information 5. [file 41598_2021_99368_MOESM5_ESM.jpg]
