# Supplementary material for: Effects of government policies on the spread of COVID-19 worldwide
Source: Sci Rep. 2021 Oct 14;11:20495. doi: 10.1038/s41598-021-99368-9 (PMC8516948; doi:10.1038/s41598-021-99368-9)
Supplement: Supplementary file 6 — Supplementary Information 6. [file 41598_2021_99368_MOESM6_ESM.docx]

**Supplementary Fig 1.** Shows the trend of daily COVID-19 Confirmed Cases for 31 Countries with one wave of the pandemic.

**Supplementary Fig 2.** Shows the trends of daily COVID-19 Confirmed Cases for 54 Countries with two waves of the pandemic.

**Supplementary Fig 3.** Shows the trends of daily COVID-19 Confirmed Cases for 5 Countries with three waves of the pandemic.

**Supplementary Fig. 4. Segmentation algorithm applied to USA’s COVID-19 daily new confirmed cases.** The blue line represents the peak and dotted sky-blue line represents breakpoint. In the 1^st^ plot, black solid line represents $\hat{f}(t)$ and black dotted line represents $Y_{t}$. The 2^nd^ plot represents cumulative confirmed cases of $Y_{t}$(black dotted line), $\hat{f}(t)$(black solid line). 3^rd^, 4^th^ plots are graphs of $\Delta\hat{f}(t), \Delta^{2}\hat{f}(t)$. In 4^th^ plot, green dotted line represents sensitivity level. If $\Delta^{2}\hat{f}(t)$ is above the upper green dotted line, $\hat{f}(t)$ is concave. On the other hand, if $\Delta^{2}\hat{f}(t)$ is below the lower green dotted line, $\hat{f}(t)$ is convex.
