# Supplementary material for: Effects of government policies on the spread of COVID-19 worldwide
Source: Sci Rep. 2021 Oct 14;11:20495. doi: 10.1038/s41598-021-99368-9 (PMC8516948; doi:10.1038/s41598-021-99368-9)
Supplement: Supplementary file 7 — Supplementary Information 7. [file 41598_2021_99368_MOESM7_ESM.docx]

**Supplementary Table 1**: A summary of the 17 government implemented policies recorded on an ordinal and numerical scale that represents the strength of the policy provided by OxCGRT. Four of the indicators (Fiscal measures, International support, Emergency investment in Healthcare and Investment in vaccines) are recorded as US dollar of fiscal spending.

| **Summary of policy variables** | | | | |
| --- | --- | --- | --- | --- |
| **ID** | **Type** | **Target/General** | **Policy name** | **Variable information** |
| C1 | Ordinal | Geographic | School closing | Policy on school and university closure sit-in and sit-in syllabus (1: Recommended for school closures, partial closures, 2: Required closures) |
| C2 | Ordinal | Geographic | Workplace closing | Policy of telecommuting or business office closure (1: closure or home recommendation, 2: partial (occupation, etc.) closure, etc.) |
| C3 | Ordinal | Geographic | Cancel public events | Public Event Cancellation Policy (1: Cancellation Recommendation, 2: Cancellation of Events) |
| C4 | Ordinal | Geographic | Restriction on gatherings | Meeting restriction policy (group restrictions of 1:1000 or more, 2:101 to 1000 participants, etc.) |
| C5 | Ordinal | Geographic | Close public transport | Public transport closure policy (1: Recommended closure, 2: Closed) |
| C6 | Ordinal | Geographic | Stay at home requirements | Outing regulation policy (1: refrain from going out, 2: outing restriction, 3: strong level of outing restrictions) |
| C7 | Ordinal | Geographic | Restrictions on internal movement | Domestic migration policy (1: Recommended staying in the local city, 2: restriction skim movers) |
| C8 | Ordinal | No | International travel controls | Response policies for foreign travelers (1: immigration checks, 2: quarantine of visitors to certain regions, etc.) |
| E1 | Ordinal | Sectoral | Income support | Financial assistance policies (1: less than 50% of existing income, 2:50% or more support) |
| E2 | Ordinal | No | Debt/contract relief | Government Debt/Contract Easing Policy (1: Certain Types of Debt Relief, 2: Total Debt/Contract Easing) |
| E3 | Numeric | No | Fiscal measures | Finances used to promote consumption, etc. |
| E4 | Numeric | No | International support | Finance used for international support for other countries |
| H1 | Ordinal | Geographic | Public Information campaign | Information on running public campaigns (1: Call attention to Covid-19, 2: Campaigns in various ways) |
| H2 | Ordinal | No | Testing policy | Criteria that can be examined (1: If you have symptoms and are satisfied with the condition, 2: if you have symptoms, etc.) |
| H3 | Ordinal | No | Contact tracing | Policy level to investigate contacts after positive confirmation (1: Investigation only in certain cases, 2: Investigation in all cases) |
| H4 | Numeric | No | Emergency investment in healthcare | Finances used for emergency health care systems |
| H5 | Numeric | No | Investment in vaccines | Finance used to develop vaccines at the national level |

| **Supplementary Table 2**: List of countries and their group used in this analysis. |
| --- |
| \| No. \| Country code \| Name \| Group \| No. \| Country code \| Name \| Group \| \| --- \| --- \| --- \| --- \| --- \| --- \| --- \| --- \| \| 1 \| AFG \| Afghanistan \| 1 \| 46 \| MRT \| Mauritania \| 1 \| \| 2 \| DZA \| Algeria \| 2 \| 47 \| MEX \| Mexico \| 1 \| \| 3 \| AUS \| Australia \| 2 \| 48 \| MAR \| Morocco \| 1 \| \| 4 \| AUT \| Austria \| 2 \| 49 \| MOZ \| Mozambique \| 1 \| \| 5 \| BHR \| Bahrain \| 2 \| 50 \| NPL \| Nepal \| 2 \| \| 6 \| BGD \| Bangladesh \| 2 \| 51 \| NLD \| Netherlands \| 2 \| \| 7 \| BLR \| Belarus \| 2 \| 52 \| NZL \| New Zealand \| 2 \| \| 8 \| BEL \| Belgium \| 2 \| 53 \| NIC \| Nicaragua \| 2 \| \| 9 \| BOL \| Bolivia \| 1 \| 54 \| NER \| Niger \| 3 \| \| 10 \| BRA \| Brazil \| 1 \| 55 \| NGA \| Nigeria \| 1 \| \| 11 \| CMR \| Cameroon \| 2 \| 56 \| NOR \| Norway \| 2 \| \| 12 \| CAN \| Canada \| 2 \| 57 \| OMN \| Oman \| 1 \| \| 13 \| CAF \| Central African Republic \| 1 \| 58 \| PAK \| Pakistan \| 1 \| \| 14 \| CHL \| Chile \| 1 \| 59 \| PER \| Peru \| 2 \| \| 15 \| CHN \| China \| 1 \| 60 \| POL \| Poland \| 2 \| \| 16 \| COG \| Congo \| 1 \| 61 \| PRT \| Portugal \| 2 \| \| 17 \| HRV \| Croatia \| 2 \| 62 \| QAT \| Qatar \| 1 \| \| 18 \| CUB \| Cuba \| 3 \| 63 \| ROU \| Romania \| 2 \| \| 19 \| CZE \| Czechia \| 2 \| 64 \| RUS \| Russia \| 1 \| \| 20 \| DNK \| Denmark \| 2 \| 65 \| SAU \| Saudi Arabia \| 1 \| \| 21 \| DJI \| Djibouti \| 3 \| 66 \| SEN \| Senegal \| 1 \| \| 22 \| ECU \| Ecuador \| 2 \| 67 \| SRB \| Serbia \| 2 \| \| 23 \| EGY \| Egypt \| 1 \| 68 \| SGP \| Singapore \| 2 \| \| 24 \| EST \| Estonia \| 2 \| 69 \| SVK \| Slovakia \| 2 \| \| 25 \| ETH \| Ethiopia \| 1 \| 70 \| SOM \| Somalia \| 1 \| \| 26 \| FIN \| Finland \| 2 \| 71 \| KOR \| South Korea \| 2 \| \| 27 \| FRA \| France \| 2 \| 72 \| SSD \| South Sudan \| 1 \| \| 28 \| GAB \| Gabon \| 2 \| 73 \| ESP \| Spain \| 2 \| \| 29 \| DEU \| Germany \| 2 \| 74 \| LKA \| Sri Lanka \| 3 \| \| 30 \| GRC \| Greece \| 2 \| 75 \| SDN \| Sudan \| 2 \| \| 31 \| GIN \| Guinea \| 2 \| 76 \| SUR \| Suriname \| 1 \| \| 32 \| HTI \| Haiti \| 1 \| 77 \| SWE \| Sweden \| 2 \| \| 33 \| HUN \| Hungary \| 2 \| 78 \| CHE \| Switzerland \| 2 \| \| 34 \| ISL \| Iceland \| 2 \| 79 \| TJK \| Tajikistan \| 1 \| \| 35 \| IND \| India \| 1 \| 80 \| THA \| Thailand \| 1 \| \| 36 \| IRQ \| Iraq \| 1 \| 81 \| TUN \| Tunisia \| 2 \| \| 37 \| IRL \| Ireland \| 2 \| 82 \| TUR \| Turkey \| 2 \| \| 38 \| ISR \| Israel \| 3 \| 83 \| UGA \| Uganda \| 2 \| \| 39 \| ITA \| Italy \| 2 \| 84 \| UKR \| Ukraine \| 1 \| \| 40 \| JAM \| Jamaica \| 2 \| 85 \| ARE \| United Arab Emirates \| 2 \| \| 41 \| JPN \| Japan \| 2 \| 86 \| GBR \| United Kingdom \| 2 \| \| 42 \| KWT \| Kuwait \| 2 \| 87 \| USA \| United States of America \| 2 \| \| 43 \| LTU \| Lithuania \| 2 \| 88 \| YEM \| Yemen \| 2 \| \| 44 \| LUX \| Luxembourg \| 2 \| 89 \| ZMB \| Zambia \| 1 \| \| 45 \| MYS \| Malaysia \| 2 \| 90 \| ZWE \| Zimbabwe \| 1 \| |
